# Efficacy, feasibility, and safety of endoscopic double closure in the GI tract

**DOI:** 10.1016/j.igie.2022.10.012

**Published:** 2022-11-04

**Authors:** Ahmad M. Al-Taee, Kohtaro Ooka, Gregory B. Haber, Jonathan Cohen

**Affiliations:** 1 Center for Therapeutic Advanced Endoscopy and Innovation, Division of Gastroenterology and Hepatology, NYU Langone School of Medicine, New York, New York, USA; 2 Kings County Hospital, Suny Downstate Medical Center, Brooklyn, New York, USA

## Abstract

Endoscopic closure is an important technique with prophylactic and therapeutic indications. In certain situations, performing a dual-modality closure might be justified over using a single-closure modality to achieve a more secure closure or to close a large defect more efficiently. Here we present 6 cases where dual-modality closure was used in a variety of indications in the upper and lower GI tracts. Efficacy was noted in all cases, and no adverse events were seen. This work highlights the feasibility, versatility, and safety of dual-modality closure using the endoscopic Helix Tacking System (X-Tack; Apollo Endosurgery, Austin, Tex, USA) to facilitate dual-modality closure in a variety of indications such as persistent gastrocutaneous fistulas and prevention of delayed bleeding after resection of high-risk lesions. Head-to-head prospective studies are needed to compare the outcomes and cost-effectiveness of dual-modality closure versus single-modality closure.

Endoscopic closure is an important technique with prophylactic and therapeutic indications such as closure of leaks and fistulas as well as the prevention of delayed bleeding after resection of high-risk lesions. In certain situations, performing a dual-modality closure might be justified over using a single-closure technique to achieve a more secure closure or to close a large defect more efficiently than using through-the-scope clips (TTSCs) alone. Here we present 6 cases in which dual-modality closure was used for a variety of indications.

## Case 1

**Figure 1:**
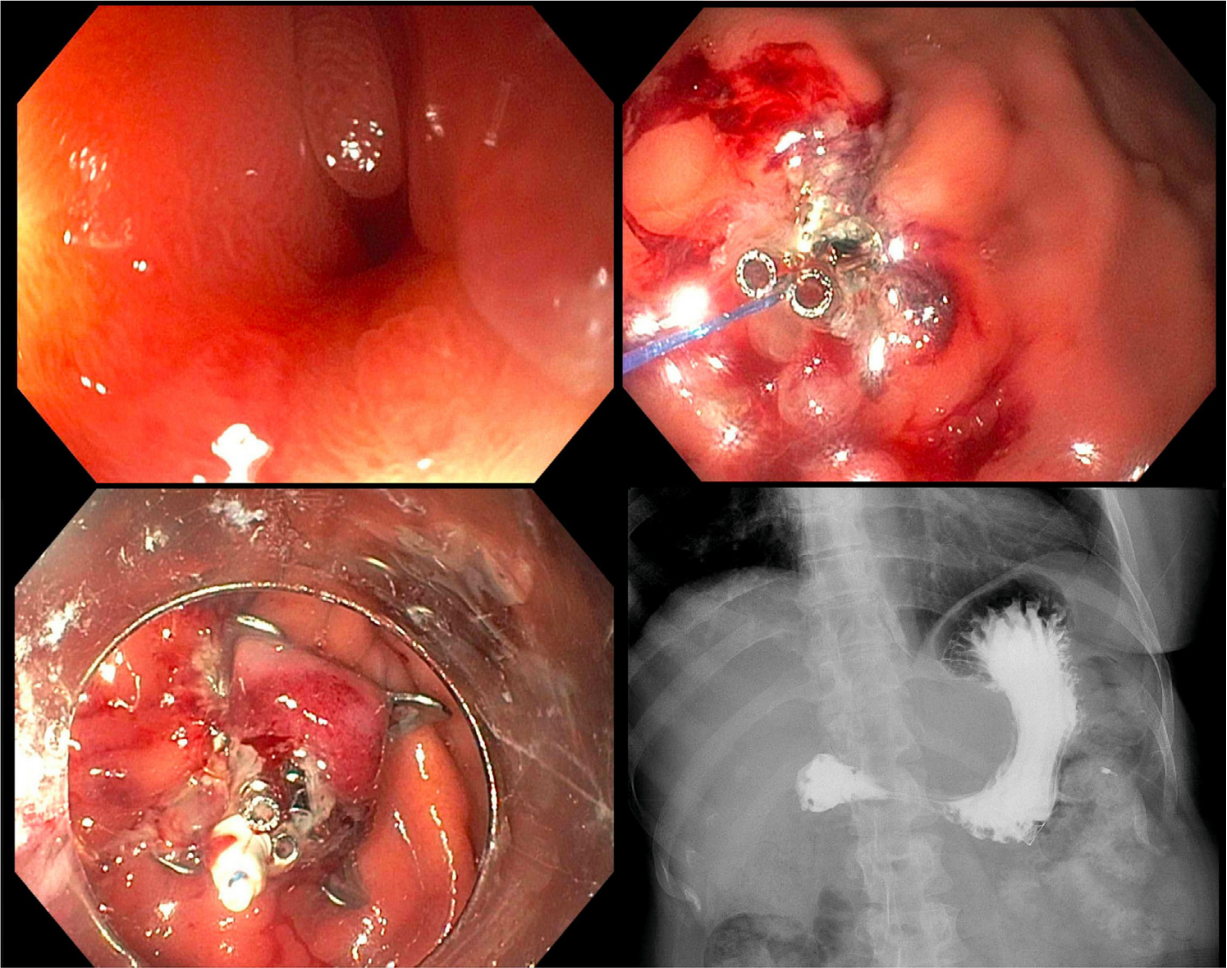
Dual-modality closure of a symptomatic persistent gastrocutaneous fistula (*top left*) using through-the-scope suturing with the endoscopic Helix Tacking System (*top right*) and an over-the-scope clip (*bottom left*). A follow-up upper GI series showed no contrast extravasation (*bottom right*).

A 56-year-old woman presented with persistent leakage from a gastrocutaneous fistula after removal of a PEG tube that did not respond to an attempt at endoscopic closure using TTSCs (Fig. 1 and Video 1, available online at www.igiejournal.org). We performed an upper endoscopy (EGD) where a single TTSC was seen at the anterior wall of the gastric body and was removed.

To de-epithelize the fistula tract, coagulation using argon plasma of the gastric side of the fistula was successful. The fistula was noted to be scarred down, which raised concern for difficulty in grabbing and pulling the tissue inside the cap of an over-the-scope clip (OTSC). To approximate the edges of the fistula and facilitate dual-modality closure, through-the-scope suturing (TTSS) using the endoscopic Helix Tacking System (X-Tack; Apollo Endosurgery, Austin, Tex, USA) was performed, and 4 tacks were deployed. An OTSC was loaded on the tip of the upper endoscope and a grasping forceps was then used to grasp the fistula and the tacks into the cap after which the OTSC was successfully deployed.

The patient tolerated fluids with no evidence of leakage and was discharged home. An upper GI series performed a week later was unremarkable, and there was no recurrent leak in 6 months of follow-up.

## Case 2

**Figure 2:**
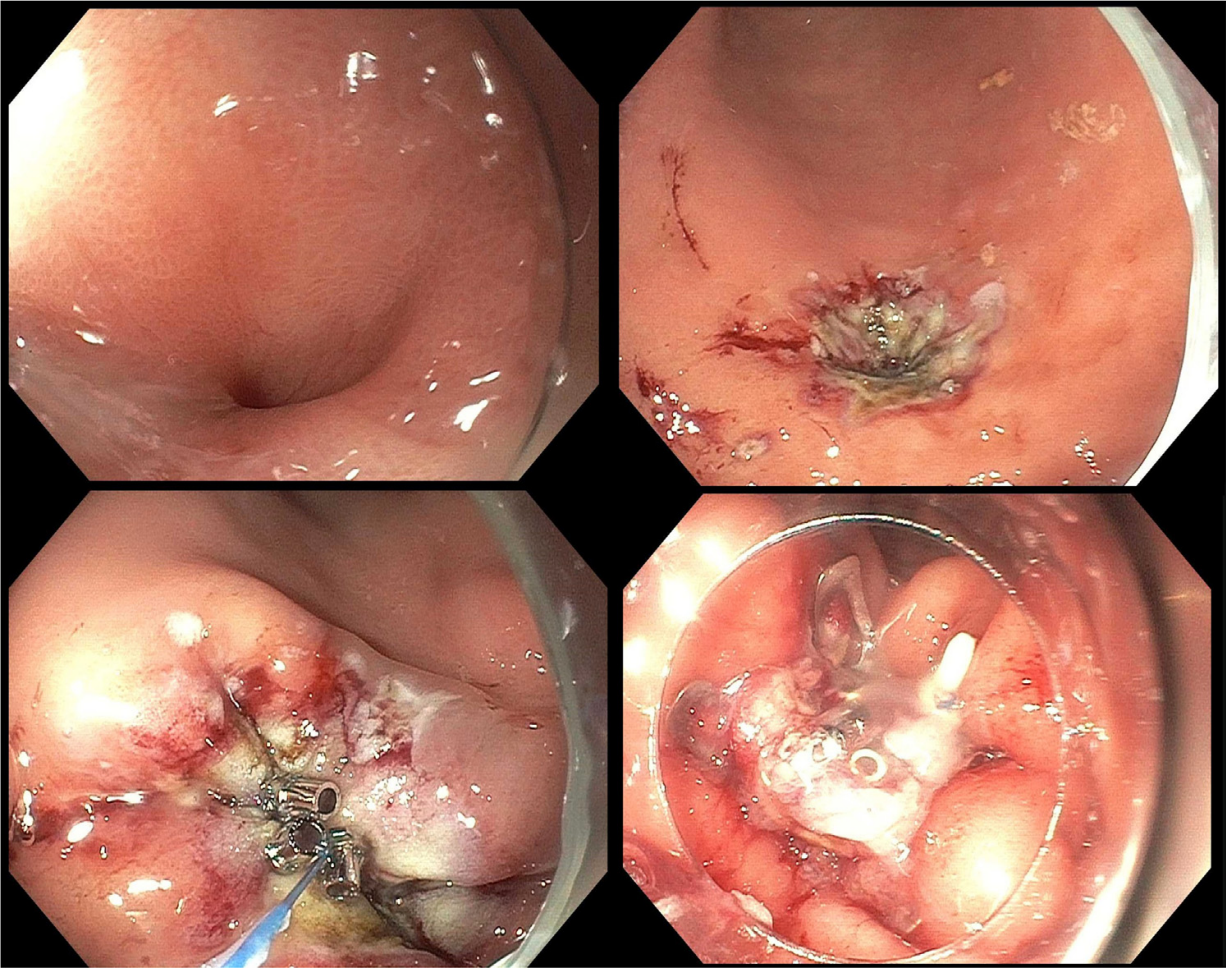
Dual-modality closure of a symptomatic persistent gastrocutaneous fistula (*top left*). After the gastric side of the fistula was treated with argon plasma coagulation to induce epithelialization (*top right*), dual-modality closure using through-the-scope suturing with the endoscopic Helix Tacking System (*bottom left*) and an over-the-scope clip (*bottom right*) was performed.

A 24-year-old man presented with persistent leakage from a gastrocutaneous fistula after removal of a PEG tube (Fig. 2). The location of the fistula was confirmed by visualizing endoscopically a biliary wire passed into the stomach from the external opening of the fistula.

The gastric side of the fistula was treated with argon plasma coagulation and brushed with a cytology brush to induce epithelialization. TTSS was used to apply a layer of sutures in a running fashion. To reinforce the closure, 1 OTSC was placed. During deployment of the OTSC, the sutures were grabbed with grasping forceps to center the fistula and to pull the defect into the cap. A leak test was performed by pooling water in a depression made by pressing down on the external fistula site. The stomach was insufflated. No bubbles were seen in the pool of water. On follow-up 3 weeks later the fistula was closed and had not leaked.

## Case 3

**Figure 3:**
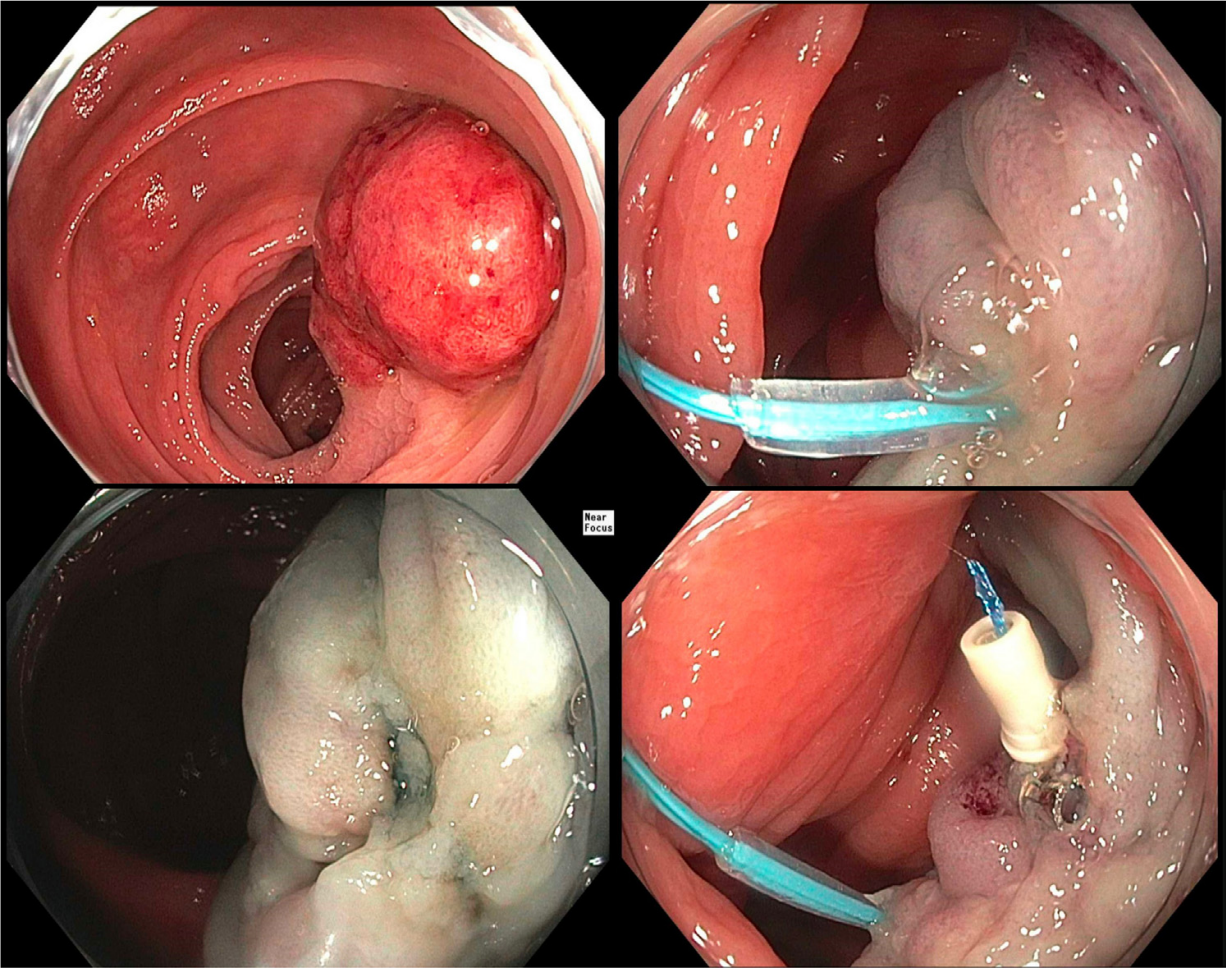
Endoscopic resection of a large ascending colon pedunculated polyp (*top left*). Dual-modality closure using the detachable loop ligating device (*top right*) and through-the-scope suturing with the endoscopic Helix Tacking System (*bottom right*) was performed.

A 47-year-old man presented for endoscopic resection of a large colon polyp found on screening colonoscopy (Fig. 3 and Video 2, available online at www.igiejournal.org). Colonoscopy revealed a 35-mm pedunculated ascending colon polyp with a stalk over 15 mm in width.

The stalk was injected with a solution of epinephrine and methylene blue. A detachable loop ligating device (Polyloop; Olympus America, Center Valley, Pa, USA) was then placed around the stalk. A snare was placed around the stalk in between the loop and neck of the polyp, and en-bloc hot snare resection using coagulation current was performed. To prevent delayed bleeding if the loop fell off the base prematurely, TTSS was used, and 4 tacks were successfully placed to close the top of the stalk.

Histologic examination revealed a tubular adenoma with a negative margin and no evidence of high-grade dysplasia (HGD) or malignancy. Surveillance colonoscopy in 3 years was recommended.

## Case 4

**Figure 4:**
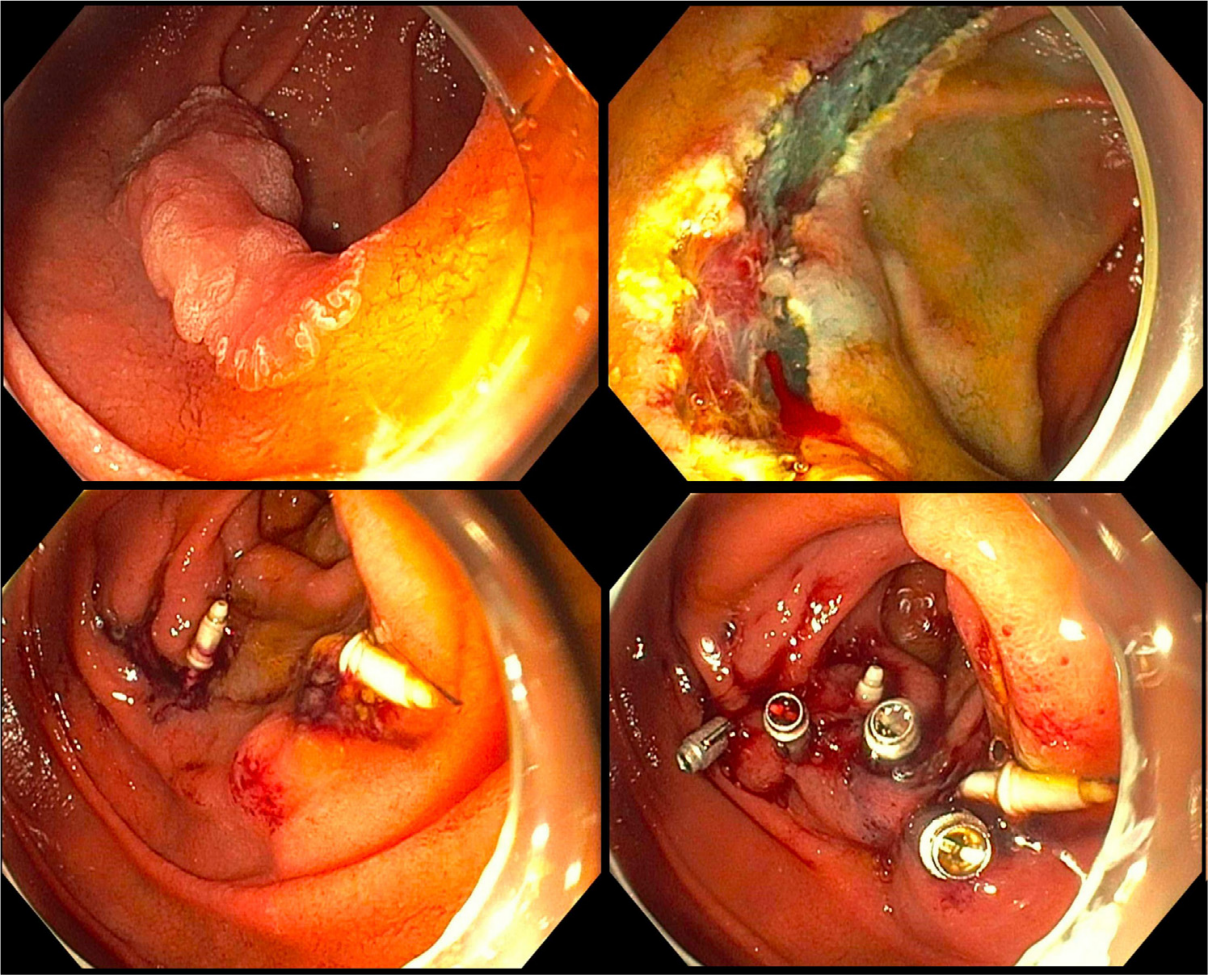
EMR of a nonampullary duodenal polyp (*top 2 panels*). A small oozing vessel in the resection base was successfully treated with soft coagulation using the closed hot biopsy forceps. Dual-modality closure using through-the-scope suturing with the endoscopic Helix Tacking System (*bottom left*) and through-the-scope clips (*bottom right*) was performed.

A 71-year-old man was referred for management of a duodenal polyp (Fig. 4 and Video 3, available online at www.igiejournal.org). EGD showed a 25-mm sessile polyp on the lateral wall of the second part of the duodenum. The major papilla was located on the opposite wall of the polyp.

Piecemeal mucosal resection using a hot snare was performed. A small oozing vessel in the resection base was successfully treated with soft coagulation using the closed hot biopsy forceps. The decision was made to perform dual-modality closure of the resection base. TTSS was used, and 8 tacks were deployed to approximate the edges of the resection base. Furthermore, 4 hemostatic clips were successfully deployed to reinforce the closure, and good tissue approximation was noted.

Histologic examination revealed a tubular adenoma and no evidence of HGD or malignancy. A follow-up EGD 6 months later revealed no residual adenoma, and 1 suture with 4 tacks remained in place.

## Case 5

**Figure 5:**
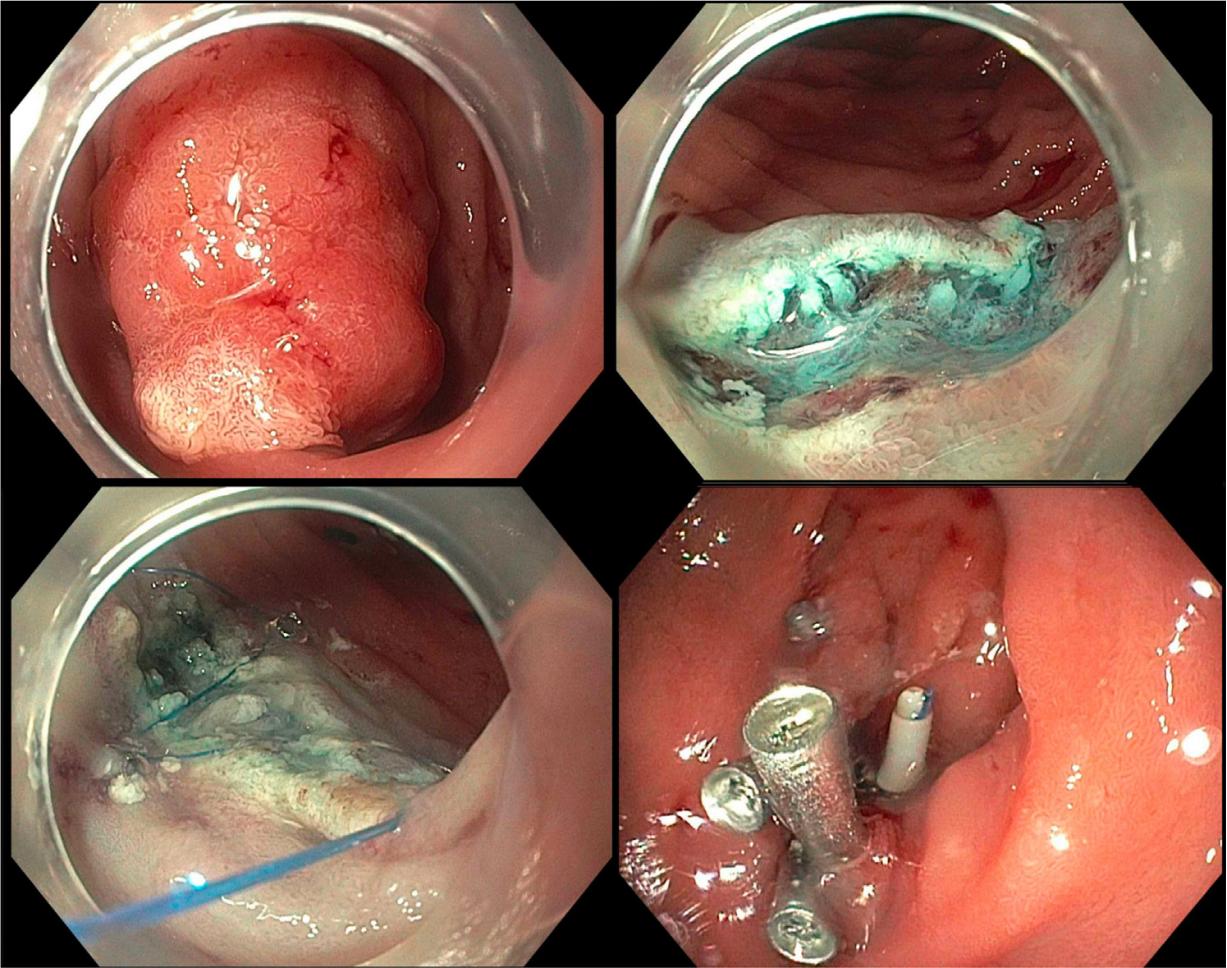
EMR of a nonampullary duodenal polyp (*top 2 panels*). Dual-modality closure using through-the-scope suturing with the endoscopic Helix Tacking System (*bottom left*) and through-the-scope clips (*bottom right*) was performed.

A 68-year-old woman with history of a duodenal polyp was referred to us for further management (Fig. 5). EGD revealed a 30-mm sessile polyp in the duodenal bulb.

Piecemeal mucosal resection was performed using hot snare. A small slowly oozing vessel at the polyp resection base was noted and was successfully treated with soft coagulation using a hot biopsy forceps. The resection defect was large and likely too wide for closure using TTSCs. Therefore, TTSS was used to approximate the edges of the resection base. Three TTSCs were then deployed with successful and complete closure of the rest of the resection base. There was no active bleeding at the end of the procedure or delayed bleeding.

Histologic examination revealed a tubulovillous adenoma and no evidence of HGD or malignancy. A follow-up upper endoscopy was scheduled for surveillance in 6 months.

## Case 6

**Figure 6:**
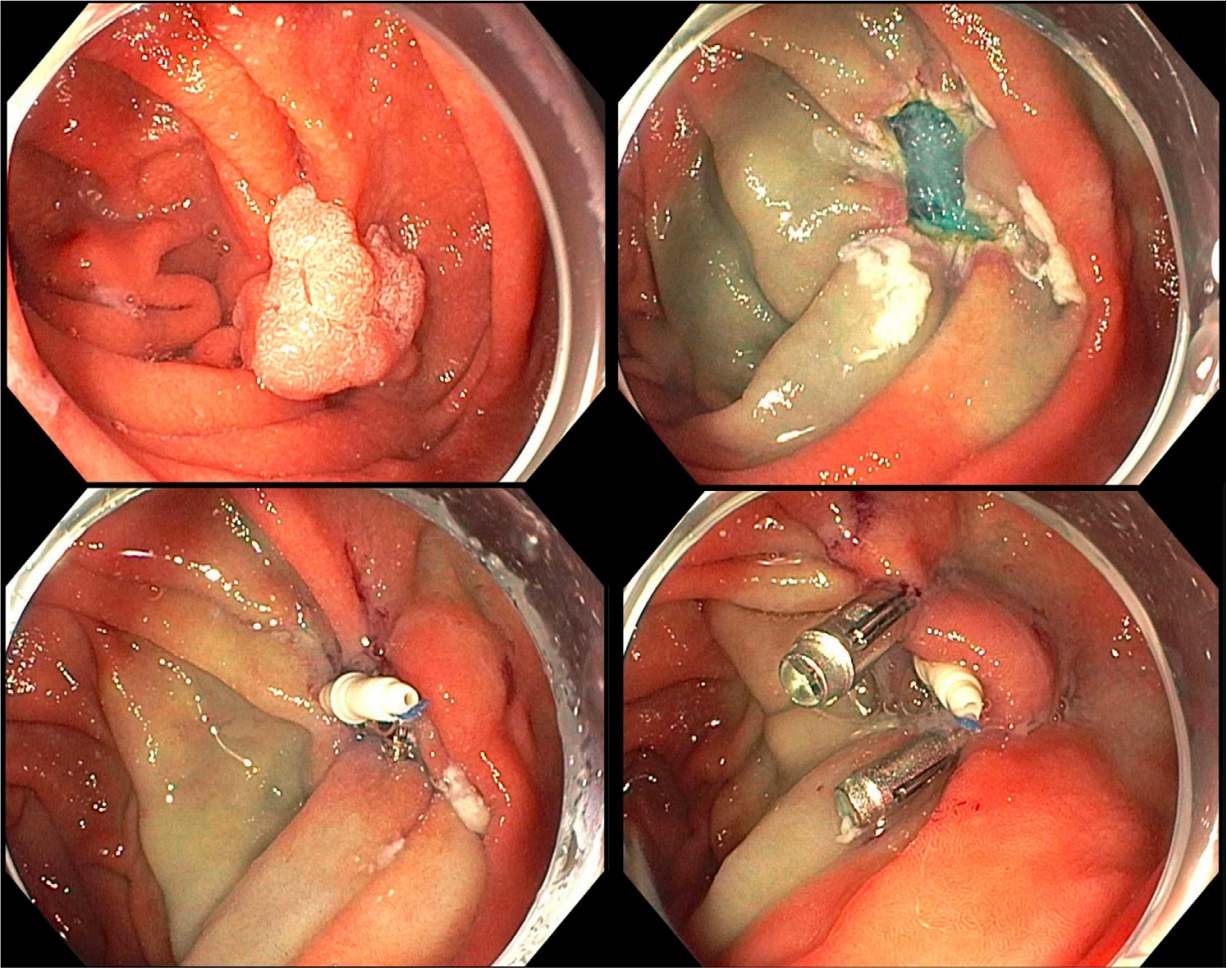
EMR of a nonampullary duodenal polyp (*top 2 panels*). Dual-modality closure using through-the-scope suturing with the endoscopic Helix Tacking System (*bottom left*) and through-the-scope clips (*bottom right*) was performed.

An 82-year-old man with history of coronary artery disease with stent placement on dual antiplatelet therapy presented for evaluation of a duodenal polyp (Fig. 6). EGD revealed a 20-mm sessile polyp in the second portion of the duodenum, distal to the major papilla.

En-bloc mucosal resection was then performed using a hot snare. The decision was made to use the TTSS system to close the resection site. Four tacks were used to close the defect with good tissue approximation of the margins. Two additional hemostatic clips were successfully placed to close the margins up the superior and inferior margins of the defect and to provide a second layer of closure. There was no active bleeding at the end of the procedure, and no delayed bleeding was noted on follow-up.

Histologic examination revealed a tubulovillous adenoma without evidence of HGD or malignancy. There was no delayed bleeding. Repeat EGD 6 months later showed a healthy-appearing mucosal resection scar with no evidence of polypoid tissue. Biopsy samples of the scar were unremarkable.

## Discussion

The presented cases demonstrate feasibility and safety of dual-modality closure in a variety of indications such as refractory nonhealing fistulas and prevention of bleeding after resection of high-risk lesions. We described the use of TTSS for 3 reasons: technical support to assist in the successful primary closure through an OTSC, efficiency concerns in closing wide defects and situations that would otherwise require a large number of TTSCs; and a combination with TTSCs to provide potentially deeper submucosa-to-submucosa approximation to offer better postpolypectomy bleeding prevention than TTSCs alone.

Persistent gastrocutaneous fistula is a rare adverse event after PEG tube removal, and endoscopic management can be challenging. Options include TTSCs, OTSCs, suturing, tissue adhesives/plugs, or a combination of these methods.^1^, ^2^, ^3^, ^4^ Given concern for scarring of the fistula tract that might affect the ability to draw the gastric side of the fistula into the cap of the OTSC, we performed dual-modality closure in which the TTSS system was used to precisely gather healthy gastric tissue together from around the fistula opening. Then, by grasping the tacks, it was easy to pull the fistula tissue into the cap followed by successful OTSC deployment. The rationale for using TTSS in this case was to facilitate the application of the primary OTSC deep closure modality.

The risk of bleeding after resection of large colonic pedunculated polyps is estimated to be about 8%.^5^ Therefore, prophylactic mechanical ligation of the polyp stalk using either a detachable loop or clips before polypectomy is recommended.^6^ It is not clear if using the 2 closure techniques is superior to either one alone.^7^ However, there have been reports of technical failure of the detachable loop.^8^ Loop slippage is a real concern if the snare resection is performed too close to the loop. To ensure this wide stalk remained securely closed would have required multiple clips applied on top of the stalk. Therefore, we believed the TTSS system was a better and more efficient option to close the top of the stalk.

Bleeding and perforation are the major adverse events of mucosal resection of nonampullary duodenal polyps. Delayed bleeding is reported in up to 15% of cases,^9^^,^^10^ and endoscopic closure is therefore performed. Closure of large and wide resection defects using TTSCs might not be feasible or might require many clips. Therefore, we used the TTSS system to approximate the edges of the resection base to a size where closure with the least number of TTSCs would be feasible. Moreover, we suspect that this approach may be cost-effective because of the smaller number of TTSCs needed after optimal approximation of edges of the resection base. The cost of 1 TTSC ranges between $150 and $370.^11^ Closure of large resection defects requires a large number of clips, and cost of closure can total to a few thousand dollars. In comparison, a single endoscopic Helix Tacking System including the cinch can cost up to $700.^12^

The TTSS system has a short learning curve and is relatively easy to set up on standard upper endoscopes and colonoscopes. Although the depth of tissue anchoring with the tacks may vary, it is generally .35-mm deep and as such may potentially secure a submucosa-to-submucosa approximation, which is deeper than the mucosal layer depth of tissue usually grabbed with TTSCs. Whether this offers an advantage over TTSC closure in prevention of delayed bleeding will require future investigation.

The cases we presented in this work highlight the feasibility, versatility, and safety of dual-modality closure using the endoscopic Helix Tacking System to facilitate closure of persistent gastrocutaneous fistulas and to prevent delayed bleeding after resection of high-risk lesions. Efficacy was noted in all cases, and no adverse events were seen. Head-to-head prospective studies are needed to compare the outcomes of dual-modality closure with single closure and the compared cost and efficacy of using TTSS versus multiple TTSCs in defect closure.

## Patient Consent

The authors have received appropriate patient consent for the publication of this article.

## Disclosure

The following authors disclosed financial relationships: G. B. Haber: Consultant for Ovesco and Olympus. J. Cohen: Consultant for Olympus and Micro-Tech; stock options in Virtual Health Partners, GI Windows, and Rom-Tech; owner of MD Medical Navigators; royalties from Wiley and Wolters Kluwer. All other authors disclosed no financial relationships.

## Supplementary data

Video 1 Dual modality closure of refractory gastrocutaneous fistula.

Video 2 Dual modality closure after resection of a large pedunculated colon polyp.

Video 3 Dual modality closure after resection of a large duodenal polyp.
